# Membrane Omega-3 Fatty Acid Deficiency as a Preventable Risk Factor for Comorbid Coronary Heart Disease in Major Depressive Disorder

**DOI:** 10.1155/2009/362795

**Published:** 2009-09-16

**Authors:** Robert K. McNamara

**Affiliations:** Department of Psychiatry, University of Cincinnati College of Medicine, Cincinnati, OH 45267, USA

## Abstract

Major depression disorder (MDD) significantly increases the risk for coronary heart disease (CHD) which is a leading cause of mortality in patients with MDD. Moreover, depression is frequently observed in a subset of patients following acute coronary syndrome (ACS) and increases risk for mortality. Here evidence implicating omega-3 (n-3) fatty acid deficiency in the pathoaetiology of CHD and MDD is reviewed, and the hypothesis that n-3 fatty acid deficiency is a preventable risk factor for CHD comorbidity in MDD patients is evaluated. This hypothesis is supported by cross-national and cross-sectional epidemiological surveys finding an inverse correlation between n-3 fatty acid status and prevalence rates of both CHD and MDD, prospective studies finding that lower dietary or membrane EPA+DHA levels increase risk for both MDD and CHD, case-control studies finding that the n-3 fatty acid status of MDD patients places them at high risk for emergent CHD morbidity and mortality, meta-analyses of controlled n-3 fatty acid intervention studies finding significant advantage over placebo for reducing depression symptom severity in MDD patients, and for secondary prevention of cardiac events in CHD patients, findings that n-3 fatty acid status is inversely correlated with other documented CHD risk factors, and patients diagnosed with MDD after ACS exhibit significantly lower n-3 fatty acid status compared with nondepressed ACS patients. This body of evidence provides strong support for future studies to evaluate the effects of increasing dietary n-3 fatty acid status on CHD comorbidity and mortality in MDD patients.

## 1. Introduction

In the year 2000, the World Health Organization (WHO) identified major depressive disorder (MDD) as the fourth ranked cause of disability and premature death globally and projected that by 2020 MDD and ischemic heart disease will be the two most important causes of disability worldwide [1, 2]. These global trends suggest that the prevalence rates of MDD and coronary heart disease (CHD) are increasing in parallel, and emerging data suggest a high degree of symptomatic comorbidity. Specifically, meta-analyses and systematic reviews of epidemiological evidence support the following: (1) clinical depression is an independent risk factor for the development of CHD, (2) severity of depression is positively correlated with CHD risk, (3) a subset of patients with CHD (20–30%) exhibit comorbid depression, (4) depressive symptoms increase secondary cardiovascular events and mortality in CHD patients, and (5) CHD is a leading cause of excessive mortality in MDD patients, particularly among women [3–9]. The high rate of CHD comorbidity in MDD is therefore suggestive of a common underlying pathoaetiological mechanism(s).

The pathoaetiology of MDD [10–12] and CHD [13–16] involves both genetic and environmental factors, and both genetic and environmental risk factors have been proposed to account for their comorbidity [17]. A rapidly emerging body of evidence suggests that membrane omega-3 (n-3) fatty acid deficiency is a preventable risk factor for both CHD [18] and MDD [19]. Based on this body of evidence, a scientific advisory panel convened by the American Psychiatric Association (APA) recommended that patients with affective disorders including MDD increase their daily EPA+DHA intake to 1 g/d [20], a daily dose previously endorsed by the American Heart Association (AHA) [21]. Although this evidence was obtained from studies conducted largely in parallel in the fields of cardiology and psychiatry, the potential contribution of n-3 fatty acid deficiency to increased CHD morbidity and mortality in MDD has been postulated previously [22, 23]. Nevertheless, a large body of evidence has emerged subsequently that permits a re-evaluation of this potential interrelationship.

To evaluate the hypothesis that n-3 fatty acid deficiency is a risk factor for CHD morbidity and mortality in MDD, it is important to consider that the age at onset for unipolar and bipolar depression peaks in young adulthood (15–19 years) [24, 25], whereas CHD mortality peaks substantially later (75–84 years) [26]. Therefore, this hypothesis would additionally posit that cerebral hypoperfusion and depressed mood [27, 28] are early manifestations of cardiovascular insufficiency associated with n-3 fatty acid deficiency [29, 30], and the progressive deterioration of cardiovascular and cerebrovascular integrity over the lifespan increases risk for CHD morbidity and mortality in older patients with MDD [31]. Accordingly, this hypothesis predicts that optimization of n-3 fatty acid status and associated cardiovascular and cerebrovascular integrity prior to onset will be an efficacious preventive strategy for both MDD and CHD.

## 2. Omega-3 Fatty Acid Biosynthesis and Membrane Accrual

**Figure 1:**
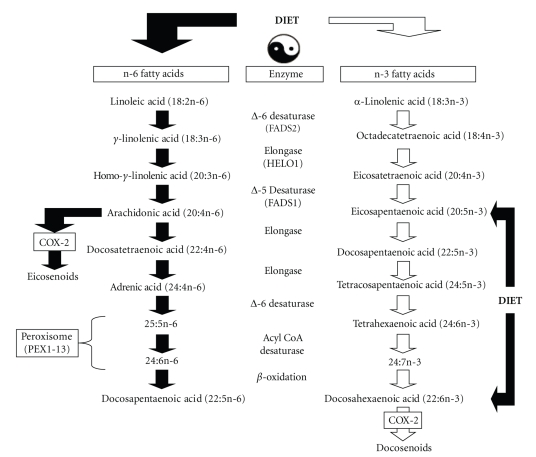
Diagram illustrating the biosynthetic pathway of n-3 and n-6 fatty acids from dietary precursors. The biosynthesis of EPA (20:5n-3) and DHA (22:6n-3) from dietary *α*-linolenic acid (18:3n-3), and arachidonic acid (20:4n-6) from linoleic acid (18:2n-6), requires a series of common and competitive microsomal elongation and delta-5 and delta-6 desaturase-mediated reactions. The final synthesis of DHA requires additional modifications within peroxisomes. Preformed DHA (22:6n-3), the principle membrane esterified n-3 fatty acid, and EPA can be obtained directly from the diet. COX-2 mediated metabolism of DHA and arachidonic acid yields anti-inflammatory docosenoids and proinflammatory eicosanoids, respectively. Because the n-6 and n-3 arms compete for common biosynthetic enzymes, elevations in the dietary LA : ALA ratio increase n-6 fatty acid biosynthesis (reflected as reductions in membrane LA and elevations in downstream fatty acid metabolites) and reduced n-3 fatty acid biosynthesis from ALA (reflected as reductions in membrane EPA+DHA).

As background, mammals are incapable of synthesizing n-3 fatty acids *de novo* and are therefore entirely dependent on dietary sources to procure and maintain adequate concentrations in peripheral and central phospholipid membranes. Dietary sources of the vegetable n-3 fatty acid precursor *α*-linolenic acid (ALA, 18:3n-3) include flaxseed, rape, linseed, canola, soy, and perilla oils. The biosynthesis of eicosapentaenoic acid (EPA, 20:5n-3) and docosahexaenoic acid (DHA, 22:6n-3) from ALA requires a series of microsomal elongation and delta-5 and delta-6 desaturase-mediated reactions [32, 33], and the final synthesis of DHA requires additional conversions within peroxisomes [34] (Figure 1). Nevertheless, preformed EPA and DHA can be obtained directly from the diet, including salmon, sardines, trout, and tuna, seafood and supplements, thereby bypassing this biosynthetic pathway. Delta-5 (*FADS1*) and delta-6 (*FADS2*) desaturase genes are colocalized to chromosome 11 (11q12-13.1), and are highly expressed in liver, heart, and brain [35–38]. The expression and activities of these enzymes are regulated by multiple factors including dietary n-3 fatty acid intake [35, 36, 39], gonadal hormones [40–42], and insulin [43]. Furthermore, the biosynthesis of n-6 fatty acids from the dietary precursor linoleic acid (LA, 18:2n-6) utilizes the same enzymatic pathway in a competitive manner (Figure 1). Accordingly, dietary deficits in ALA (18:3n-3) are associated with a significant increase in n-6 fatty acid biosynthesis from dietary LA [44, 45], and western diets which have a high LA content are associated with limited (<1.0%) EPA and DHA biosynthesis from ALA [46–50].

Emerging data also indicate that common single nucleotide polymorphisms in *FADS1* and *FADS2* genes are an important determinant of RBC, plasma, and breast milk n-3 and n-6 fatty acid composition [51–54]. For example, a case-control study found that subjects carrying *FADS* haplotypes and consuming a Western diet exhibit a higher index of n-6 fatty acid biosynthesis (arachidonic acid : LA ratio), and this index was an independent risk factor for coronary artery disease [55]. Taken collectively, these findings indicate that membrane n-3 and n-6 fatty acid composition is governed by both environmental (dietary n-6/n-3 fatty acid ratio) and genetic factors.

## 3. Epidemiology

There are large cross-national variations in per capita fish/seafood consumption, a surrogate for EPA+DHA intake. For example, annual seafood consumption in Japan (148 lb/person) is approximately 3-fold higher than in the USA (48 lb/person) [56], and this is reflected in a ~2-fold greater red blood cell (RBC) membrane EPA+DHA composition in Japanese adults (8.5 ± 1.8%) [57] compared with USA adults (4.9 ± 2.1%) [58]. Moreover, there are large cross-national variations in the lifetime prevalence rates of MDD and CHD mortality. For example, life-time prevalence rates of MDD in Japan (<3%) [59, 60] are ~6-fold lower than in the USA (16.2%) [61], and the prevalence of CHD mortality among Japanese men aged 55–64 years (58/100 000) is 6-fold lower than among American white men in the USA (345/100 000) [62]. Across multiple countries, there is a significant inverse correlation between per capita fish or seafood consumption and the lifetime prevalence rates of unipolar depression [63, 64], bipolar depression [65], and postpartum depression [66]. Together, these data suggest that cross-national variations in n-3 fatty acid intake are inversely correlated with the prevalence of MDD and CHD mortality. However, the potential contribution of other nutrients found in fish/seafood and other cultural and genetic differences preclude definitive evaluation of their interrelationship. 

On a smaller scale, cross-sectional studies have also observed an inverse relationship between dietary n-3 fatty acid intake and prevalence rates of MDD, and that this relationship is stronger among women [67–72]. For example, the Northern Finland 1966 birth cohort study, a prospective survey of 2721 males and 2968 females adults (age: 31 years), found that the risk of developing depression increased 2.6-fold among females, but not males, who were rare fish eaters (monthly) compared with regular (weekly) fish eaters [72]. Moreover, a cross-sectional survey of 9806 male and 12029 female adult and elderly subjects (40–74 years) from Norway found that subjects who ingested cod liver oil on a daily basis (DHA: ~300–600 mg/d, EPA: ~300–600 mg/d) were significantly less likely to have depressive symptoms than nonusers after adjusting for multiple confounding factors [70]. Similarly, cross-sectional surveys have found an inverse correlation between dietary n-3 fatty acid intake and CHD risk factors, nonfatal acute coronary syndrome (ACS), and sudden cardiac death (SCD) [73, 74]. Lastly, shifting diets in Arctic populations away from fish-based to western diets have been associated with increased rates of both depression and CHD [75].

## 4. Prospective Longitudinal Observational Studies

Another approach taken to evaluate the contribution of n-3 fatty acid status to the pathoaetiology of MDD and CHD is prospective longitudinal observational studies using baseline dietary n-3 fatty acid intake or membrane n-3 fatty acid composition as the predictor variable. In one of the largest prospective studies conducted to date, EPA+DHA intake was determined at baseline in 3317 young adult men and women (mean age: 32 years) residing in the USA, and depressive symptoms determined at a 10-year follow-up. It was found that among both men and women, the highest quartile of EPA+DHA intake at baseline was associated with a lower adjusted risk for depression at 10 years relative to the lowest quintile, and this association was stronger in women [76]. Other studies have evaluated membrane n-3 fatty acid status and the emergence of perinatal depression in pregnant women. In a recent prospective case-control study of depressed and nondepressed women, it was found that higher plasma DHA and total n-3 fatty acid composition in the third trimester were associated with a significantly lower adjusted risk for depression [77]. In this study, women with the lowest plasma n-3 fatty acid levels were six times more likely to be depressed than women who had the highest n-3 fatty acid levels. Similarly, another study found that low or no n-3 fatty acids intake from fish/seafood were associated with a higher incidence perinatal depression relative to the highest quartiles of n-3 fatty acids intake [78]. A third study found that faster recovery of plasma DHA content postpartum was associated with reduced risk of postpartum depression [79]. Together, these findings provide prospective evidence for an inverse and independent relationship between n-3 fatty acid status and the emergence of clinical depression.

Several large prospective longitudinal studies have evaluated the relationship between baseline n-3 fatty acid status and the emergence of CHD mortality. In the Physicians' Health Study, a prospective case-control study of 14,916 healthy adult male physicians (aged 25–74 years) found that 94 men experienced sudden cardiac death (SCD) over the following 17 years. Whole blood long-chain n-3 fatty acid composition in SCD cases was compared with 184 demographically matched controls. It was found that the adjusted risk for SCD was reduced by 80% in subjects with the highest blood n-3 fatty acid levels compared with those with the lowest levels [80]. In a similar study, 334 adult (aged 25–74 years) patients with primary cardiac arrest were compared with 493 control cases and controls free of prior CHD. It was found that subjects with a red blood cell (RBC) EPA+DHA composition of 3.3% exhibited a 70% greater adjusted risk for primary cardiac arrest compared with those with a RBC EPA+DHA composition of 5.0% [81]. Similarly, in a cohort of 94 subjects with ACS and 94 age-, gender-, and race-matched controls, whole blood EPA+DHA composition in ACS patients was 29% lower than in controls, and the adjusted odds ratio for ACS was 0.67 [82]. The Nurses' Health Study, a prospective analysis of 84688 women (aged 34–59 years) without evidence of prior CHD and followed for up to 16 years, found that women who rarely ate fish (<1 per month) had a higher risk of CHD death compared with women with a higher intake of fish, with adjusted relative risks (RR) of 1.0, 0.93, 0.78, 0.68, and 0.67 across intake quintiles [83]. Lastly, a meta-analysis of 13 cohorts including 222364 subjects followed for a mean of 11.8 years found that subjects with low or no n-3 fatty acids intake from fish/seafood experienced greater CHD mortality compared with individuals with a higher intake of fish [84]. The adjusted RRs for CHD mortality decreased with increasing fish intake frequency: 1–3 times per month (RR: 0.89), once per week (RR: 0.85), 2–4 times per week (RR: 0.77), and 5 or more times per week (RR: 0.62). Each 20g/d increase in fish intake was related to a 7% decreased risk of CHD mortality. 

Together, these data indicate that low or no EPA+DHA intake is an independent risk factor for both CHD and MDD. However, only one prospective longitudinal study has evaluated the interrelationship between baseline n-3 fatty acid intake, baseline depressive symptom severity, and the emergence of CHD. The Zutphen Elderly Study, a prospective cohort study conducted in 332 elderly men (aged 70–90 years) residing in the Netherlands, found that higher baseline dietary EPA+DHA intake (mean: 407 mg/d) was associated with a 54% lower adjusted risk for mild to severe depression relative to lower dietary EPA+DHA intake (mean: 21 mg/d). However, baseline dietary EPA+DHA intake was not associated with a significant difference in the adjusted risk for CHD mortality during the 10-year follow-up period [85]. The authors concluded that these data do not support the hypothesis that lower n-3 fatty acid intake can account for the relationship between depression and CHD.

## 5. Controlled n-3 Fatty Acid Intervention Trials

A method used to evaluate the causal relationship between n-3 fatty acid status and the pathophysiology of MDD and CHD is double blind randomized placebo-controlled n-3 fatty acid intervention trials. The results of randomized controlled n-3 fatty acid intervention trials in patients with unipolar or bipolar depression have been systematically reviewed previously [19, 20, 86–90]. Despite small sample sizes, negative trials, heterogeneity in study design in terms of daily n-3 fatty acid dose, type of n-3 fatty acid intervention (DHA, EPA, EPA+DHA), EPA : DHA ratio, intervention duration, concomitant medication effects, and depression symptom severity at baseline, independent meta-analyses have found a significant advantage of n-3 fatty acid treatment over placebo in patients with clinical depression [20, 86, 87, 90]. Importantly, the majority of patients in these trials were being administered antidepressant or mood-stabilizer medications, which may have blunted the effect size associated with the n-3 fatty acid intervention. For example, a preliminary randomized, double-blind placebo-controlled trial found that chronic dietary n-3 fatty acid (EPA+DHA) *monotherapy* significantly reduced symptom severity in pediatric and adolescent (8–12 years) patients with MDD [91]. Independent meta-analyses have also found a significant advantage of n-3 fatty acid treatment over placebo or usual care for event-free survival in patients with established CHD [92, 93]. In one of the largest studies conducted to date, the Japan EPA Lipid Intervention Study found that the risk for nonfatal major cardiac events in a cohort of 18645 hypercolecterolemic men and women with or without a history of CHD was reduced by 19 percent in patients treated with EPA plus statin compared with statin alone [94]. Together, these secondary prevention studies provide evidence that depression symptom severity and CHD risk are both significantly attenuated by increasing n-3 fatty acid status. To date, however, there have been no controlled n-3 fatty acid primary prevention (monotherapy) trials conducted in subjects at risk for CHD or MDD, nor have there been primary prevention trials evaluating cardiac outcomes in MDD patients.

## 6. Membrane n-3 Fatty Acid Composition Studies

Case-control studies have compared the n-3 fatty acid status of patients with demographically similar healthy controls using plasma, RBC, and adipose n-3 fatty acid composition. Importantly, RBC membrane EPA+DHA composition is highly correlated with habitual dietary EPA+DHA intake and plasma phospholipid EPA+DHA composition [95, 96]. Moreover, RBC membrane EPA+DHA composition is highly correlated human myocardium biopsy EPA+DHA composition [97] and primate frontal gray matter biopsy DHA composition [98]. Based on prior prospective longitudinal data reviewed above, RBC EPA+DHA composition (termed the “omega-3 index”) has been proposed as a novel risk biomarker for CHD mortality, and risk zones have been defined [99]. Specifically, RBC EPA+DHA composition of <4% is high risk, 4%–8% is intermediate risk, and >8% is low risk. An “omega-3 index” of ≤4% is associated with a 10-fold greater risk for SCD compared to an omega-3 index of >8% [99]. Additionally, RBC EPA+DHA composition is significantly lower in patients with acute coronary syndrome (ACS) compared with age-, sex-, and race-matched controls [100], and a meta-analysis of prospective studies found that RBC DHA composition was inversely correlated with CHD events [101]. 

**Figure 2:**
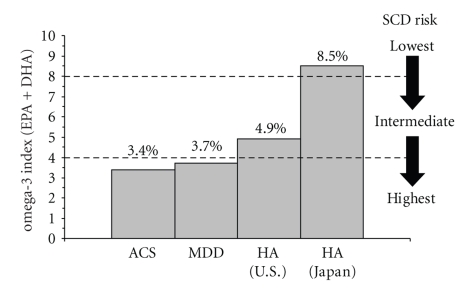
Comparison of the “omega-3 index” (RBC EPA+DHA composition) in adult USA patients with acute coronary syndrome (ACS, *n* = 768) [100], adult MDD patients residing in the USA or UK (*n* = 42) [102, 105, 106], healthy adults (HAs) residing in the U.S. (*n* = 163) [58], and healthy adults residing in Japan (*n* = 456) [57]. Proposed risk zones for sudden cardiac death (SCD) derived from prior prospective longitudinal evidence are indicated [99]. Note that MDD patients exhibit an “omega-3 index” that is similar to patients with ACS and places them at high risk for SCD.

In the last 20 years there have been at least 6 published case-control studies from developed western countries that have compared peripheral (plasma, RBC) n-3 fatty acid composition in MDD patients and healthy controls. In all of these studies, significantly lower plasma or RBC EPA+DHA composition was observed in MDD patients relative to healthy controls [102–107] (Table 1). In a small case-control study conducted in the USA, we recently found that young adult MDD patients (*n* = 17, mean age: 35 years) exhibited RBC EPA+DHA composition that was significantly lower than healthy adult controls (*n* = 20, mean age: 35 years) (3.8% ± 1.0% versus 4.8 ± 1.1%, *P* = .006) [105]. In this cohort, 65% of MDD patients exhibited an RBC EPA+DHA index of ≤4.0% compared with 25% of controls. Analysis of combined case-control studies indicates that MDD patients (*n* = 548) exhibit RBC or plasma EPA+DHA values that are 21% lower than healthy controls (*n* = 218) (3.9 ± 0.4% versus 5.0 ± 0.8%, *P* = .01) (Table 1). These findings are significant because they suggest that patients with MDD exhibit an RBC EPA+DHA index that is similar to that observed in patients with ACS (3.4 ± 1.6%) [100] and, based on prospective observational studies, suggests that at least a subset of MDD patients are in the high-risk (≤4.0%) category for SCD [99]. Specifically, these data suggest that MDD patients have a ~60% greater risk for primary cardiac arrest compared with healthy controls [81] and a 10-fold greater risk for SCD compared to adult Japanese subjects (Figure 2). 

To date there have been three studies that have determined the interrelationship between n-3 fatty acid status and depression in patients with established CHD. In the first study, ACS patients diagnosed with MDD exhibited significantly lower plasma EPA+DHA levels compared with nondepressed ACS patients [108]. In a second study, ACS patients with current depression had significantly lower mean plasma total n-3 fatty acid and DHA compared with those without current depression, and depression symptom severity was inversely correlated with plasma DHA composition [109]. In a third larger study, among ACS patients (*n* = 759), those with depression exhibited significantly lower RBC EPA+DHA composition relative to those without depression (2.9 ± 1.5% versus 3.3 ± 1.8%, *P* = .002) [110]. Taken collectively, these data support an inverse relationship between n-3 fatty acid status and depression in ACS patients.

## 7. Biological Mechanisms

There are several plausible biological mechanisms that could potentially link membrane n-3 fatty acid deficiency observed in MDD with increased risk for CHD. In addition to emerging clinical and preclinical evidence that n-3 fatty acids are protective against cardiac arrhythmias [111–114], other mechanisms including enhanced platelet reactivity and aggregation, elevated triglyceride levels, and inflammation, have received increasing experimental attention and are reviewed separately below.

### 7.1. Platelet Aggregation

The antithrombotic actions of n-3 fatty acids have been well documented [115], and platelet functional abnormalities, including increased platelet reactivity and aggregation associated with thromboembolic events, have been proposed as a potential mechanism by which depression increases risk for CHD [116, 117]. Blood platelets express serotonin 5-HT_2A_ receptors, and elevated 5-HT_2A_ receptor binding density has been observed in platelets, as well as postmortem prefrontal cortex, of depressed suicide victims [118–122]. Moreover, chronic dietary n-3 fatty acid insufficiency is associated with significant elevations in 5-HT_2A_ receptor binding density in the rat prefrontal cortex [123, 124]. Platelet 5-HT_2A_ receptors are coupled to the phosphoinositide (PI) signal transduction pathway, and platelets obtained from MDD patients exhibit several indices consistent with PI-signaling hyperactivity, including elevated basal and/or stimulated intracellular inositol triphosphate, diacylglycerol, and/or calcium concentrations [125]. It is relevant, therefore, that previous preclinical studies have found that chronic EPA+DHA feeding significantly reduces indices of basal and stimulated PI signal transduction in platelets, neutrophils, and cardiac myocytes [126–129]. Moreover, 5-HT_2A_ receptors may also be coupled to phospholipase A_2_ (PLA_2_) which liberates membrane acetylated arachidonic acid, a precursor for proinflammatory prostaglandin synthesis, and chronic dietary n-3 fatty acid deficiency is associated with significant elevations in PLA_2_ activity in the rat prefrontal cortex [130]. Together, these data suggest that elevated platelet 5-HT_2A_ receptor-mediated signaling secondary to n-3 fatty acid deficiency may increase risk of thromboembolic events in patients with MDD and CHD [131].

### 7.2. Inflammation

The mechanisms by which EPA+DHA reduce inflammatory signaling have been reviewed in detail previously [132] and include direct competition for cyclooxygenase-2- (COX-2) mediated arachidonic acid metabolism into proinflammatory prostaglandins and suppression of proinflammatory cytokine production. Several cross-sectional studies have observed an inverse correlation between blood EPA+DHA levels and markers of inflammation, including C-reactive protein (CRP) and proinflammatory cytokine interleukin-6 (IL-6) levels, among healthy adult men and women [133–136]. Inflammation is thought to be a key factor in the progression of atherosclerotic vascular disease, and elevations in the inflammatory marker CRP are associated with higher long-term risk for future CHD events [137]. Moreover, a cross-sectional study of 992 individuals with stable coronary artery disease found that RBC DHA+EPA levels were inversely and independently correlated with CRP and IL-6 levels [138], and a randomized controlled study found that chronic (91 d) DHA treatment significantly reduced CRP and IL-6 levels in hypertriglyceridemic men [139]. Cross-sectional surveys and case-control studies have also found elevated blood markers of inflammation, including CRP and IL-6, in patients with MDD relative to healthy controls, and that these markers were positively correlated with depression symptom severity [140–145]. Lastly, greater CRP levels have been observed in CHD outpatients with MDD compared with CHD outpatients without MDD [146]. However, it is notable that the Heart and Soul Study of 984 participants found that current MDD was associated with *lower* levels of inflammatory marker including CRP and IL-6 in both unadjusted and adjusted models [147].

### 7.3. Elevated Triglycerides

Elevated triglyceride levels (200–500 mg/dL) have been found to be an independent, albeit modest, risk factor for CHD [148–150]. It has been known for over 20 years that the regular consumption of fish is associated with a 20%–40% reduction in plasma triglyceride levels [151], and n-3 fatty acids decrease hepatic triglyceride biosynthesis and hepatic clearance [152]. Several randomized controlled trials have consistently observed significant reductions in triglyceride levels (~25%) following daily EPA+DHA (~4 g/d) treatment relative to placebo in patients with hypertriglyceridemia (≥500 mg/dL) [153]. It is also notable that a study reported reduced depression symptom severity following resolution of severe hypertriglyceridemia (≥500 mg/dl) after treatment with gemfibrozil (Lopid) [154]. The American Heart Association currently recommends that patients with hypertriglyceridemia consume 2–4 g EPA+DHA per day [155], and in 2004 the U.S. Food and Drug Administration approved EPA+DHA ethyl esters (Omacor) for adjunctive treatment of hypertrigyceridemia. Moreover, two randomized controlled trials found that concomitant treatment with EPA [156] or EPA+DHA [157] significantly and dose-dependently decreased elevated triglyceride levels in schizophrenic patients treated with the atypical antipsychotic clozapine. 

Surprisingly few studies have investigated fasting triglyceride levels in patients with MDD, and the existing results are mixed. For example, one small case-control study found that although fasting triglyceride levels in male and female MDD patients did not differ from healthy controls, triglyceride levels were greater in male patients with melancholic features compared with men with atypical features [158]. A second case-control study that did not segregate by MDD subtype did not observe significant differences in fasting triglyceride levels in MDD patients (*n* = 100) relative to healthy controls (*n* = 100) [159]. Moreover, a cross-sectional study of 3490 men aged 31–45 years did not observe elevated triglycerides in subjects diagnosed with MDD [160], and another study found that triglyceride levels were not associated with depression 1 month after an acute myocardial infarction [161]. However, a cross-sectional study found that in a sample of 5439 men aged 71–89 years, 32% of subjects with depression had high fasting triglyceride levels compared with 21% of nondepressed subjects [162], and patients diagnosed with MDD after a recent ACS exhibited significantly higher fasting triglyceride levels relative to nondepressed ACS patients [108]. Although these findings do not unanimously support elevated triglyceride levels in MDD, recent studies have found a stronger association between nonfasting (postprandial state) triglyceride levels and cardiovascular risk compared to fasting triglyceride levels [163, 164]. Moreover, elevated triglyceride levels (>150 mg/dL) are observed in 25%–35% of the general U.S. population [165]. Future studies are therefore warranted to investigate whether patients with MDD exhibit elevated nonfasting triglyceride levels, and whether these levels are associated with n-3 fatty acid status and depression symptom severity.

## 8. Conclusions and Hypothesis Evaluation

There is now a substantial body of evidence linking n-3 fatty acid deficiency with both MDD and CHD. Support for the hypothesis that n-3 fatty acid deficiency represents a preventable risk factor for comorbid CHD in MDD includes (1) cross-national and cross-sectional epidemiological surveys finding an inverse correlation between n-3 fatty acid status and prevalence rates of both CHD and MDD, (2) prospective studies finding that lower dietary or RBC EPA+DHA levels increase risk for both MDD and CHD, (3) case-control studies finding that a large subset of MDD patients exhibit an RBC EPA+DHA level that places them at high risk for emergent CHD morbidity and mortality relative to the general population, (4) meta-analyses of randomized controlled secondary n-3 fatty acid intervention studies finding significant advantage of n-3 fatty acids over placebo for secondary prevention of cardiac events in CHD patients and depression symptom severity in MDD patients, (5) cross-sectional and controlled intervention studies finding that n-3 fatty acid status is inversely correlated with other documented CHD risk factors, including increased platelet reactivity and aggregation, augmented inflammatory signaling, and elevated fasting triglyceride levels, and (6) ACS patients diagnosed with MDD exhibit significantly lower plasma EPA+DHA levels compared with nondepressed ACS patients. 

The most direct refutation of this hypothesis comes from a prospective longitudinal study finding that baseline n-3 fatty acid intake was inversely correlated with baseline depressive symptoms but was not associated with increased long-term CHD risk [85]. However, this study has several notable limitations. Firstly, this study did not account for potential changes in dietary n-3 fatty acid intake after baseline measurements were obtained, and it remains possible that subjects increased n-3 fatty acid intake postbaseline. Second, this study was conducted in a relatively small cohort (*n* = 332), and larger prospective trials have observed an inverse relationship between baseline n-3 fatty acid status and long-term CHD risk (reviewed above). Lastly, this study used a dietary n-3 fatty acid questionnaire to define baseline n-3 fatty acid status, and no objective measures (i.e., RBC EPA+DHA composition) were collected to corroborate accuracy. 

In view of the weight of current evidence, additional studies are clearly warranted to directly evaluate the interrelationship between n-3 fatty acid deficiency and CHD comorbidity in MDD. A definitive test of the proposed hypothesis would be to determine in a prospective longitudinal controlled trial whether increasing the n-3 fatty acid status of MDD patients decreases indices of CHD risk and emergent cardiac events and mortality. Increasing RBC EPA+DHA composition to ≥8%, which is associated with the greatest protection from CHD mortality [99], would represent an appropriate target, and a prior study demonstrated that RBC DHA+EPA composition of 7.8% was achievable after 8-week treatment with DHA (864 mg/d) + EPA (1,296 mg/d) (~2.0 g/d) in adult male and female subjects [166]. Older MDD patients at greater risk for CHD mortality at study entry (i.e., RBC EPA+DHA ≥4.0%) would represent an appropriate cohort. Based on the evidence reviewed here, it is predicted that increasing n-3 fatty acid status of MDD patients to ≥8% would be associated with a significant reduction in CHD risk factors, including depression symptom severity, nonfasting triglyceride levels, indices of platelet aggregation, CRP and IL-6 levels, and ultimately reduced risk for emergent cardiac events and SCD.

## Figures and Tables

**Table 1:** Omega-3 index in patients with MDD.

Study	Country	Blood fraction	Sample size (case : control)	Omega-3 index^1^ (case : control)	Δ
Maes et al. [103]	Belgium	Plasma	36 : 24	3.6 : 3.8	−5%
Maes et al. [104]	Belgium	Plasma	34 : 14	4.5 : 4.9	−7%
Tiemeier et al. [107]	Netherlands	Plasma	106 : 461	4.4 : 4.6	−4%
Peet et al. [106]	U.K.	RBC	15 : 15	3.6 : 6.3	−42%
Edwards et al. [102]	U.K.	RBC	14 : 10	3.8 : 5.4	−31%
McNamara et al. [105]	U.S.	RBC	17 : 20	3.8 : 4.8	−21%
Total	—	—	218 : 548	3.9 : 5.0	−21%

Δ = Mean percent difference from controls

^1^Mean EPA+DHA wt % total fatty acid composition.
